# Clinical Tools to Assess and Monitor Spondyloarthritis

**DOI:** 10.1007/s11926-015-0522-3

**Published:** 2015-06-12

**Authors:** Robert Landewé, Astrid van Tubergen

**Affiliations:** Amsterdam Rheumatology & Immunology Center, Academic Medical Center, Amsterdam, The Netherlands; Atrium Medical Center, Heerlen, The Netherlands; Department of Medicine, Division of Rheumatology, Maastricht University Medical Center, Maastricht, The Netherlands

**Keywords:** Spondyloarthritis, SpA, Axial spondyloarthritis, axSpA, Core set, Index, Spinal mobility, Physical function, Peripheral joint, Sacro-iliac joint, Spine, Visual analog scale, Numerical rating scale, Enthesitis, Arthritis, Sacroiliitis

## Abstract

Assessment and monitoring is important in diseases affecting multiple sites and organs, such as axial spondyloarthritis (axSpA) that may have several signs and symptoms, and for which several treatments are available. Instruments for assessment and monitoring should be appropriately validated, and it should be feasible to use them in clinical practice as well as in clinical trials. The Assessment in SpondyloArthritis International Society (ASAS) has developed core-sets of domains of disease and instruments to measure these domains, and recommends only the most important domains being measured with the best available methods. This article describes the ASAS core sets, as well as a few recent developments in the field of assessment, to be applied in clinical practice and research studies.

## Introduction

Spondyloarthritis, or better axial spondyloarthritis (axSpA) in the context of this article, includes non-radiographic axSpA (with clinical signs and symptoms of SpA, but without characteristic radiographic changes on pelvic X-rays) and radiographic axSpA (synonymous to ankylosing spondylitis (AS)). AxSpA is a ‘system-disease’, with inflammation as its main hallmark, that may affect many sites and structures of the human body, and therefore may give a multitude of various signs and symptoms. Each of these symptoms can be assessed in clinical practice by a variety of instruments, but it is obvious that this multitude does not contribute to a better understanding of the disease, especially in the context of patient care.

In order to create parsimony [1], the Assessment in SpondyloArthritis International Society (ASAS) has established a core set of variables to be formally measured in patients with AS [2, 3]. Since symptoms and burden of disease of nr-axSpA and AS do not importantly differ, ASAS has proclaimed that this core set of variable is similarly applicable to the entire spectrum of axSpA.

In this article, domains and instruments available for the assessment and monitoring of nr-axSpA will be briefly discussed in the context of the ASAS core set. In addition, disease activity indices and response measures (reported in clinical trials) will be mentioned.

## The ASAS Core Sets

This ASAS/OMERACT core set of outcome measures spells out the minimum of variables that should be collected for three different settings: “symptom-modifying anti-rheumatic drugs” (SM-ARDs) and physical therapy, clinical record keeping and “disease-controlling anti-rheumatic treatment” (DC-ART).

SM-ARDs are non-steroidal anti-inflammatory drugs (NSAIDs). DC-ARTs in AS are tumor necrosis factor-alpha (TNF-α) inhibitors and sulfasalazine.

**Fig. 1:**
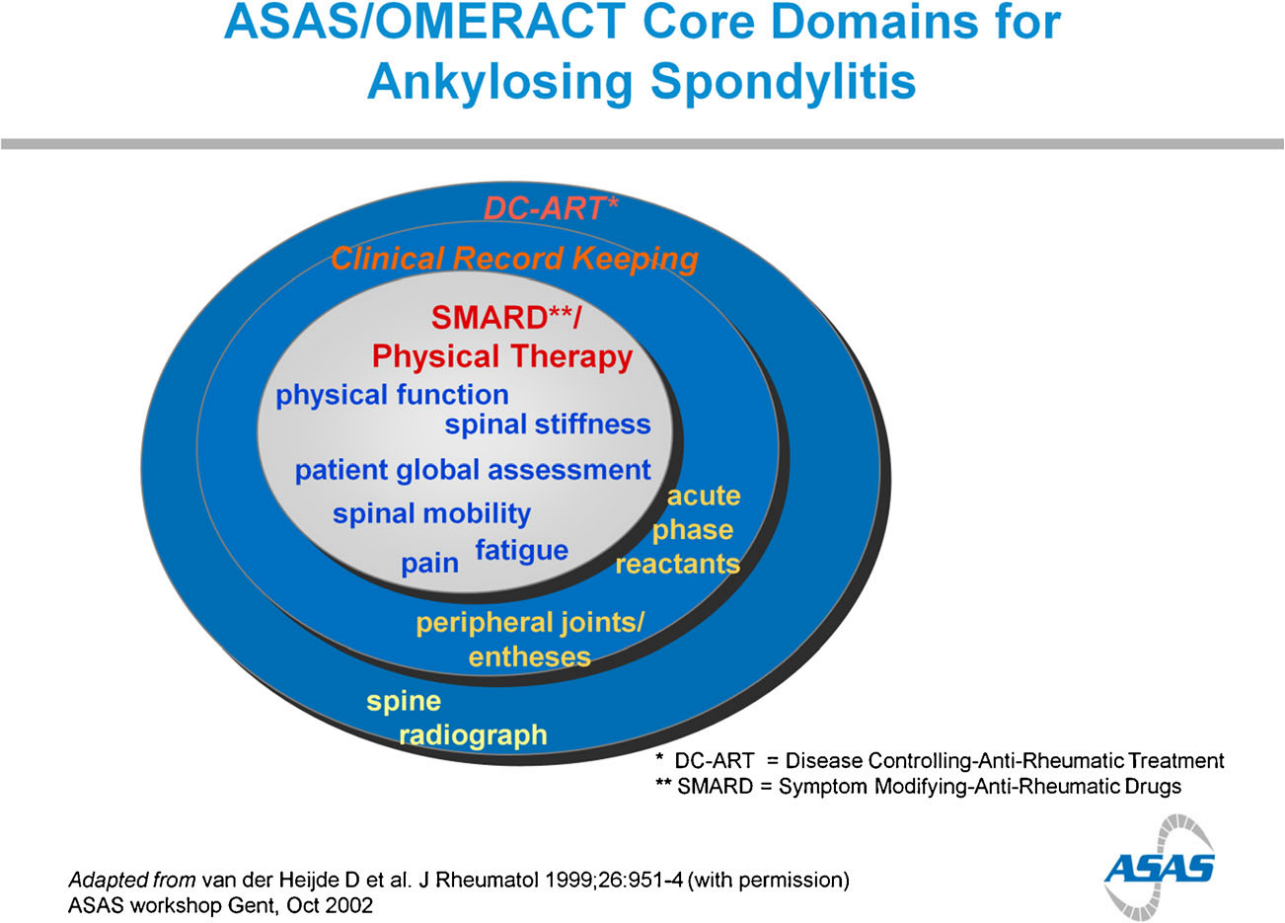
ASAS/OMERACT core domains for ankylosing spondylitis

**Fig. 2:**
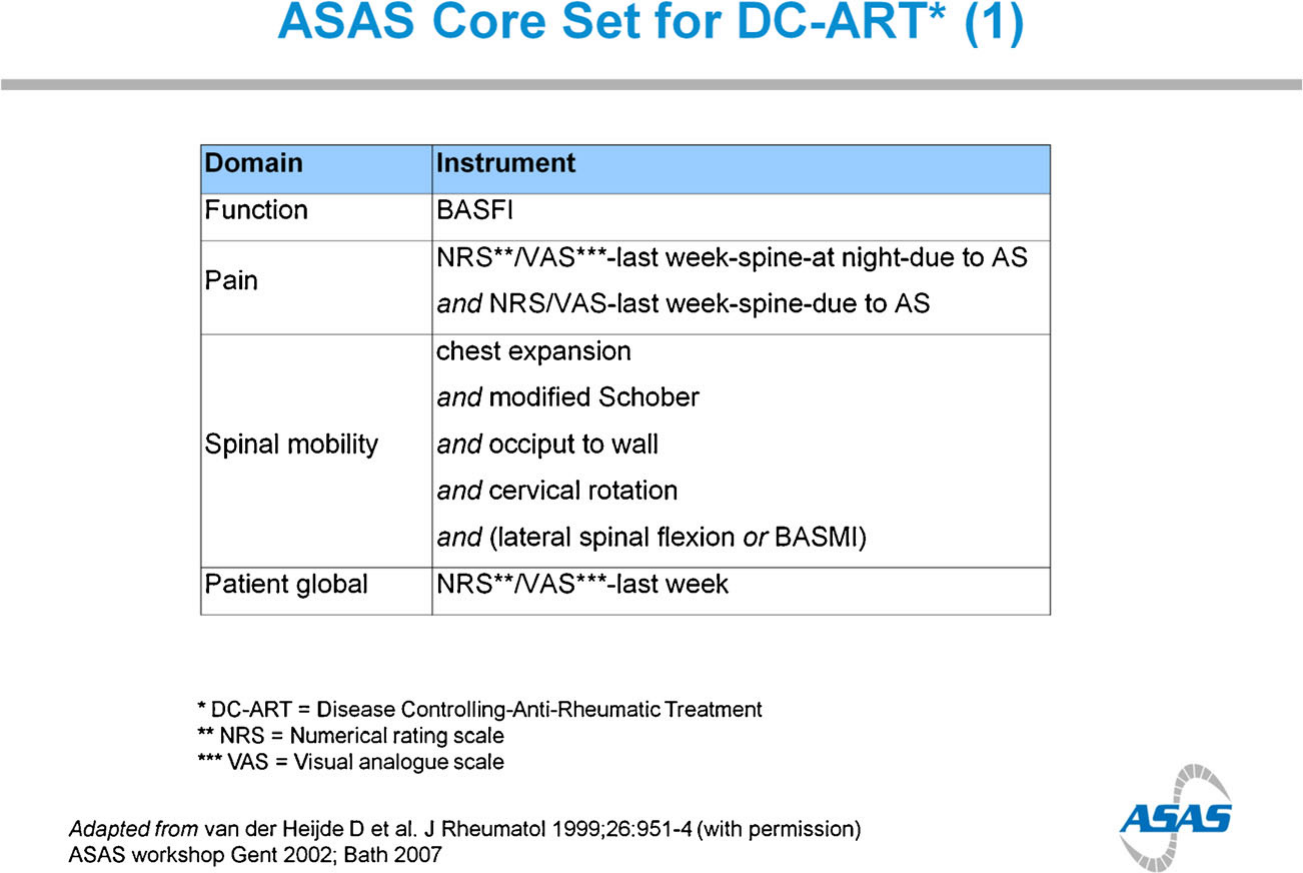
ASAS Core Set for DC-ART* (1)

The core set includes the relevant domains, but specific instruments have been selected for each domain (Figs. 1 and 2).

### Core Set of Symptom-Modifying Anti-rheumatic Drugs and Physical Therapy

The inner circle of the ASAS/OMERACT core set describes the domains that should be assessed in patients receiving SM-ARDs and/or physical therapy. Specific instruments to assess each of these domains are described below and can be found on the website of the ASAS: www.ASAS-group.org

#### Physical Function

Physical function is an important outcome in axial SpA. Two indices are used to measure function in axial SpA: the Bath AS Functional Index (BASFI) and the Dougados Functional Index (DFI) [4, 5]. BASFI includes 10 questions; 8 of them refer to aspects of functional anatomy, and 2 pertain to the ability to cope with everyday’s life. All questions are completed on numerical rating scales (NRS) or on a 10 cm visual analog scale (VAS) with “easy” and “impossible” as anchors. The total BASFI-score is the average of the 10 questions and ranges from 0 to 10.

The DFI is the second index that measures function in axial SpA [6]. To date, the DFI is hardly ever used and no longer recommended for clinical practice nor research.

#### Spinal Stiffness

“How long does your morning stiffness last from the time you wake up?” This simple question is supposed to give insight into the inflammatory symptom “spinal stiffness”. The answer is preferably recorded on an NRS or alternatively on a 10 cm VAS with three anchors: “0 h,” “1 h,” and “2 or more hours”. Scores range from 0 (none) to 10 (severe).

#### Patient Global Assessment

As in every rheumatologic condition, patient global assessment is considered relevant in axSpA and assessed by the question: “How active was your spondyloarthritis last week?” The answer is recorded on a NRS or on a VAS, and the score ranges from 0 (not active) to 10 (very active).

#### Spinal Mobility Measurement

Many instruments have been developed to assess spinal mobility. ASAS has positively recommended to assess chest expansion, modified Schober’s test, occiput-to-wall distance (OWD), cervical rotation, and lateral spinal flexion [2, 3]. As an alternative for lateral spinal flexion, also the Bath AS Metrology Index (BASMI) can be used [7]. The BASMI is an index that combines five measures, slightly different as recommended above: tragus-to-wall distance (TWD), modified Schober’s test, cervical rotation, lateral spinal flexion, and intermalleolar distance. The measures will be briefly described here, since there is often confusion about the optimal methodology. Descriptions below are derived from the ASAS handbook, to be checked on www.ASAS-group.org

- Chest expansion (only included in ASAS/OMERACT core set)
  The patient is asked to rest his hands on or behind the head. The difference between maximal inspiration and expiration is measured at the fourth intercostal level anteriorly in cm to the nearest 0.1 cm. Chest expansion is measured twice, and the better of two tries is recorded.
- Modified Schober (included in ASAS/OMERACT core set and BASMI)
  The patient is standing erect. An imaginary line, connecting both posterior superior iliac spines, is marked. A second mark is placed 10 cm above the first mark. The patient is asked to bend forward maximally, and the distance between the two marks is measured. The increase in cm to the nearest 0.1 cm is recorded. The modified Schober is measured twice, and the better of two tries is recorded.
- Occiput to wall distance (included in ASAS/OMERACT core set) and tragus to wall distance (included in BASMI)
  The patient is standing with the heels and back resting against the wall, with the hips and knees as straight as possible. The chin should be held at the usual carrying level. The patient is asked to put maximal effort to touch the head against the wall. The distance between occiput and wall is measured in cm to the nearest 0.1 cm. The OWD is measured twice, and the better of two tries is recorded.
- The TWD is measured with the patient in the same position as the OWD measurement. The distance between the tragus and wall is measured twice in cm to the nearest 0.1 cm on the left side, and the better of two tries is recorded. The same procedure is followed for the right side. The final TWD is calculated by averaging the best value for the left and the right side.
  Both the OWD and TWD were found to be equally reliable in assessing thoracic spine extension [8]. A limitation of the TWD is that it can vary with the size of the head. However, the TWD is less influenced by inclination of the cervical spine. The OWD is recommended by ASAS, because (and in contrast with TWD) an OWD value of 0 distinguishes a patient with a “normal” thoracic spine extension from a patient with in kyphosis due to axSpA [8].
- Cervical rotation (included in ASAS/OMERACT core set and BASMI)
  The patient is sitting straight on a chair, and the chin at the normal carrying level. The assessor places a goniometer at the top of the head in line with the nose. The patient is asked to rotate the neck maximally to the left, and the assessor follows with the goniometer. The angle between the first sagittal plane and the new plane after rotation is measured in degrees. The better of two tries is recorded. The same procedure is followed for the right side. The final score for cervical rotation is calculated by averaging the best values for the left and the right side and recorded in degrees.
- Lateral spinal flexion (included in ASAS/OMERACT core set and BASMI)
  The patient is standing with the heels and back resting against the wall, without flexion in the knees and without bending forward. A first mark is placed on the right thigh, at the level of the patient’s middle fingertip. The patient is then asked to bend sideward to the right as far as possible without bending the knees or lifting the heels, and a second mark is placed again at the level of the patient’s middle fingertip. The distance between the two marks is measured in cm to the nearest 0.1 cm. The better of two tries is recorded. The same procedure is followed for the left side. The score for lateral spinal flexion is calculated by averaging the best values for the left and the right side.
- Intermalleolar distance (only included in BASMI)
  The patient is lying with the legs separated as far as possible with the knees straight and the toes pointing upwards. The distance between the medial malleoli is measured in cm. Alternatively, the intermalleolar distance is measured with the patient standing erect and the legs separated as far as possible. The better of two tries is recorded.
- BASMI
  The originally developed BASMI is called BASMI-2. All 5 continuous measurements were converted into a three-point scale (0 to 2) using a conversion table [7]. The final BASMI-2 score is calculated by summing the individual converted scores and ranges from 0 to 10. Since converting a continuous scale into a discrete scale with only 3 categories loses valuable information, a more extensive scale with 11 categories called the BASMI-10 was investigated and outperformed the BASMI-2 [9].
  A further extension of BASMI-10 was the BASMI-linear, in which the continuous scores are converted into a linear scale using five equations [10]. The BASMI-linear score is calculated by the mean of the five linear scores and ranges from 0 to 10. The BASMI-linear has shown to be more sensitive to change in clinical trials compared with the BASMI-2 and BASMI-10 [11]. Nowadays, ASAS recommends to use either the BASMI-10 or the BASMI-linear. Importantly, scores from the BASMI-10 and BASMI-linear are not interchangeable with the BASMI-2.

#### Pain

Pain is usually assessed in axial SpA by two standard questions: (a) “How much pain of your spine due to AS do you have?” and (b) “How much pain of your spine due to AS do you have at night?” The answers are recorded on a NRS or a VAS with two anchors “no pain” and “most severe pain” at each side, respectively. Score ranges from 0 (no pain) to 10 (most severe pain).

#### Fatigue

“How would you describe the overall level of fatigue/tiredness you have experienced?” is how fatigue according to ASAS should be inquired in patients with axSpA. Scores (either on NRS or on VAS) range from 0 (none) to 10 (very severe).

### Core Set of Clinical Record Keeping

The core set of clinical record keeping includes the same domains as the core set of SM-ARDs and/or physical therapy but adds the domains: “peripheral joints,” “entheses,” and “acute phase reactants”.

#### Peripheral Joints

Peripheral joint involvement in axSpA is frequent and can be assessed using the 44-joint count. Presence of swelling should be recorded, and the total score varies from 0 to 44.

#### Entheses

Enthesitis is a typical characteristic of axSpA and means inflammation at these sites [12]. The first instrument for assessing enthesitis in axSpA is known as the Mander Enthesitis Index (MEI) [13]. Essentially, the MEI quantifies the patient’s response to pain following local pressure to 66 entheseal sites and is scored on a Likert scale (0, no pain; 1, mild tenderness; 2, moderate tenderness; or 3, tenderness severe enough to elicit a wince or withdrawal). Alternatively, more parsimonious indices have been proposed. These include the San Francisco Enthesitis Index (SFI) [14], the Berlin Enthesitis Index (BEI) [15], and the Maastricht AS Enthesitis Score (MASES) [16]. These instruments were found to reliably reflect enthesitis in axSpA [17] and are included in the ASAS/OMERACT core set for DC-ARTs. The SFI examines 17 sites with a scoring identical to the MEI [18]. The BEI examines 12 sites [15]. Enthesitis is scored as 0 (absent) or 1 (present), which avoids an important part of inter-observer variation. The MASES includes 13 sites and only takes values per site of 0 (absent) or 1 (present) [16]. Other validated enthesitis indices include the Spondyloarthritis Research Consortium of Canada (SPARCC) Enthesitis Index (16 sites) and the Leeds Enthesitis Index (LEI) (6 sites) [19, 20] but are not (yet) included in the ASAS core sets. In general, ASAS does not recommend the use of enthesitis indices in daily clinical practice.

#### Acute Phase Reactants

Acute phase reactants are considered relevant in the core set of clinical record keeping. An elevated erythrocyte sedimentation rate (ESR) or C-reactive protein (CRP) is only present in 30–40 % of the patients, and it is good to realize that a normal value does not preclude the presence of inflammation. If there is peripheral joint involvement or inflammatory bowel disease present in conjunction with axSpA, the acute phase reactants are elevated more often [21]. Some suggest the use of ESR or CRP for monitoring disease flares, or to predict a more favorable response to treatment [22].

### Core Set of Disease-Controlling Anti-rheumatic Treatments

The core set of DC-ARTs includes the same domains as the core set of clinical record keeping and adds the domain “spine radiograph”.

#### Radiographic Assessment of the Spine

A few scoring methods have been proposed for assessing radiographic damage of the spine in axSpA. The best validated one, and the one recommended by ASAS, is the modified Stoke AS Spine Score (mSASSS) [23]. In the mSASSS, the anterior parts of the cervical and lumbar spine at a lateral view are scored for the presence of squaring and/or erosion and/or sclerosis (1 point per site), non-bridging syndesmophytes (2 points per site) and/or bridging syndesmophytes (3 points per site or 6 points per vertebral unit). The total score ranges from 0 to 72.

## Disease Activity Indices

Disease activity indices are increasingly popular and relevant for monitoring disease activity in patients with axSpA, but not yet included in the ASAS core set, since they are methodologically inferior [Bath AS Disease Activity Index (BASDAI)] or simply too recent [AS Disease Activity Score (ASDAS)]. The BASDAI [24] and the ASDAS [25] are the most important indices available for clinical practice.

- BASDAI
  The BASDAI includes six questions on fatigue (1), spinal pain (2), peripheral joints (3), entheses (4), intensity of morning stiffness (5), and duration of morning stiffness(6) [24]. Each question is scored on an NRS or on a 10 cm VAS, and the final BASDAI score is calculated by summing the first four questions and the average of the last two questions, and divide the result by five. The score ranges from 0 (no disease activity) to 10 (very active disease). A cutoff of 4 is frequently used to define active disease, but this cutoff level does not have a firm justification. An important drawback of the BASDAI is that it is entirely expert-driven. As a consequence, many of the six items in the BASDAI are redundant (measuring the same construct). Advantages of the BASDAI are that it is easy to complete and very well known.
- ASDAS
  The ASDAS is a data-driven index, partially based on consensus, that combines questions (patient reported outcomes (PROs)) about back pain (1), peripheral pain/swelling (2), and duration of morning stiffness (3) (1–3 are derived from the BASDAI-questions), as well as the “patient global assessment of disease activity” (4), with either the ESR (ASDAS-ESR) or the CRP (ASDAS-CRP)(5) in a weighted manner [25]. The ASDAS can be calculated as follows:
  ASDAS-CRP: $$ \begin{array}{l}0.121 \times \mathrm{total}\ \mathrm{back}\ \mathrm{pain} + 0.110 \times \mathrm{patient}\ \mathrm{global} + 0.073 \times \mathrm{peripheral}\ \mathrm{pain}/\mathrm{swelling}\hfill \\ {} + 0.058 \times \mathrm{duration}\ \mathrm{of}\ \mathrm{morning}\ \mathrm{stiffness} + 0.579 \times \mathrm{L}\mathrm{n}\left(\mathrm{C}\mathrm{R}\mathrm{P}+1\right).\hfill \end{array} $$
  ASDAS-ESR: $$ \begin{array}{l}0.113 \times \mathrm{patient}\ \mathrm{global} + 0.293\ \mathrm{x}\sqrt{\mathrm{ESR}} + 0.086 \times \mathrm{peripheral}\ \mathrm{pain}/\mathrm{swelling}\hfill \\ {} + 0.069 \times \mathrm{duration}\ \mathrm{of}\ \mathrm{morning}\ \mathrm{stiffness} + 0.079 \times \mathrm{total}\ \mathrm{back}\ \mathrm{pain}.\hfill \end{array} $$
  The ASDAS-CRP is recommended by ASAS, both for use in clinical practice and in clinical trials, but the ASDAS-ESR may be used as well. Importantly, ASAS has formally validated cutoff levels for disease activity states: an ASDAS value below 1.3 is considered low disease activity, between 1.3 and 2.1 as moderate disease activity, between 2.1 and 3.5 as high disease activity, and above 3.5 as very high disease activity, with no maximum [26••].

## Criteria for Response and Remission

Response criteria are intended to measure a response to treatment. The ASAS-defined improvement criteria as well as BASDAI and ASDAS can be used for defining improvement or response in both clinical practice and in studies.

- ASAS 20 improvement criteria
  The well-known “ASAS 20 improvement criteria” include four domains: patient global, pain, function (assessed by BASFI), and inflammation (mean of BASDAI questions 5 and 6) [27]. In order to meet an ASAS 20 response, three of the four domains should improve by at least 20 % and a minimum of one unit on a scale of 0 to 10. In the remaining domain, there should be no worsening of 20 % and a minimum of 1 unit, on a 0 to 10 scale.
- ASAS 40 improvement criteria
  ASAS 40 improvement criteria include four domains, identical to the ASAS 20 improvement criteria [28]. In order to meet an ASAS 40 response, three of the four domains should improve by at least 40 % and a minimum of two units on a scale of 0 to 10. In the remaining domain, there should be no worsening of 20 % and a minimum of 1 unit, on a 0 to 10 scale.
- ASAS 5/6 improvement criteria
  The ASAS 5/6 improvement criteria include six domains: patient global, pain, function (assessed by BASFI), inflammation (mean of BASDAI questions 5 and 6), CRP, and spinal mobility (assessed by lateral spinal flexion) [28]. In order to meet an ASAS 5/6 improvement, there should be an improvement of at least 20 % in at least five of these six domains.
- ASAS partial remission
  ASAS has defined a state of partial remission, which reflects very low disease activity [28]. In order to fulfill an ASAS partial remission state, a value of 2 (on a 0 to 10 scale) or less should be present in each of the following domains: patient global, pain, function (BASFI), and inflammation (mean of BASDAI questions 5 and 6).
- BASDAI 50 response
  ASAS has published a consensus statement for the use of TNF-α inhibitors in patients with AS in clinical practice [29]. Response to TNF-α inhibitors is defined by improvement of at least 50 % in the BASDAI score or an absolute change of 2 units (on a 0 to 10 scale) after 3 months of treatment with TNF-α inhibitors, together with an expert opinion compatible with improvement.
- ASDAS improvement criteria
  ASAS has also defined ASDAS-based response criteria. According to the ASDAS improvement criteria, a change in the score of at least 1.1 units is equivalent to a “clinically important improvement,” and a change of at least 2.0 units is called a “major improvement” [26••].
- ASDAS inactive disease
  ASAS has defined cutoffs for disease activity states, using the ASDAS score [26••]. A value below 1.3 is considered “inactive disease”.

## ASAS Health Index

Important complaints of patients with axSpA are, as said, pain, fatigue, and limitation in activities and social participation. Thus far, the instruments that have been discussed here for the assessment of patients with SpA focus predominantly on specific aspects of health such as pain, disease activity, and physical function and measure specific concepts like physical function. However, the overall picture of impairments, limitations, and restrictions in activities or social participation of patients with AS is not adequately assessed in SpA-specific questionnaires. Most questionnaires are not conceptualized with regard to their underlying construct. The International classification of functioning, disability, and health (ICF) Core Set for AS has served as an appropriate template for developing a health index, since the whole range of functioning and disability of patients with axSpA is captured. Based on these assumptions, ASAS has developed an instrument assessing health as operationalized by the ICF for patients with SpA. This questionnaire was developed using an item pool, linkage of the items to the comprehensive ICF core set for AS and testing of the item pool. The ASAS HI is a linear composite index with 17 items, which cover most aspects of the ICF core set [30••]. Preliminary validity has been confirmed in a field test in four English-speaking countries. The ASAS HI will be used in clinical trials and in clinical practice to test its real life performance and to confirm that this new composite index captures relevant information on functioning and health of patients with axSpA.

## Conclusions

Axial spondyloarthritis is a disease with multiple facets. It is heterogeneous in its presentation, symptomatology, and course. Rather than being associated with one unequivocal outcome measure, axSpA has many “outcome faces”. All these outcomes can be measured, thus leaving the clinician and researcher with residual uncertainty about the true value of these measures. ASAS has brought parsimony in this multitude of outcome measures by prioritizing aspects of validity and feasibility (core-sets), and has also developed new instruments (e.g., ASDAS and ASAS-HI) that will help to create better understanding of all aspects of axial spondyloarthritis.
